# Supplementary material for: Patterns of Intron Gain and Loss in Fungi
Source: PLoS Biol. 2004 Nov 30;2(12):e422. doi: 10.1371/journal.pbio.0020422 (PMC532390; doi:10.1371/journal.pbio.0020422)
Supplement: Table S1 — Also available at http://genes.mit.edu/NielsenEtAl/. (4.3 MB ZIP). [file pbio.0020422.st001.zip › NielsenEtAl/html/1330.html]

AN6146.1.NCU08552.1.MG07392.1.FG06530.1


```
 CLUSTAL W (1.82) Multiple Sequence Alignments - Introns Inserted


Sequence 1: NCU08552.1	127 aa
Sequence 2: MG07392.1	131 aa
Sequence 3: FG06530.1	131 aa
Sequence 4: AN6146.1	119 aa
Alignment Length: 131 aa
Number Identitical Residues: 62 aa
Alignment Score (without introns) 3197


MG07392.1 	MIQLK0SMLNCIDNSGAALVECALIVGQKRHASIG1DRIV~VVVQEQRKNPNEGAAALAA
NCU08552.1	MIQLK0TMLNCIDNSGAALVECAMVVGQKRHASI-~---V1VVVQKQRGADSAGMAASSA
FG06530.1 	MIQLK0TMLNCIDNSGAALVECVLVVGQKRHARIG1DRVV~VVVQEQRGSSSGGMAGMSA
AN6146.1  	MIQLK0TLLNCIDNSGAAVVECVNVLKKKRAATVG1DRIV~IVVQKQRNFGPEGTSSTGI
          	***** ::**********:***. :: :** * :. .  * :***:**     * :. . 

MG07392.1 	SNKVKRGDIRHAIVVRTKYKVQRKDGSVVRFDDNACVLINKAGDPVGSRVNGIVGMELRK
NCU08552.1	ATKVKRGDIRHAVVVRTKQKVQRRDGSVVRFDDNACVLINKAGDPIGSRINGVVGQELRK
FG06530.1 	ANKVKRGDIRHAVVVRTRYPTQRPDGSIVKFDDNACVLLNKAGDPVGSRINGAVGAELKR
AN6146.1  	ANKVRRGDIRHAVVVRVKKEMQRPDGTLVKFDDNACVLVNKSGDPIGTRMN--------E
          	:.**:*******:***.:   ** **::*:********:**:***:*:*:*        .

MG07392.1 	~AKWSKILSMAPMHI
NCU08552.1	~KKWSKILSMAPMQA
FG06530.1 	~KKWSKILSMAPMQA
AN6146.1  	1HTFPGISLIT----
          	  .:. *  ::
```
